# Crystal structure of ammonium (3,5-di­chloro­phen­oxy)acetate hemihydrate

**DOI:** 10.1107/S2056989015016345

**Published:** 2015-09-12

**Authors:** Graham Smith

**Affiliations:** a Science and Engineering Faculty, Queensland University of Technology, GPO Box 2434, Brisbane, Queensland 4001, Australia

**Keywords:** crystal structure, phen­oxy­acetic acid herbicides, tryptaminium salt, (3,5-di­chloro­phen­oxy)acetic acid, hydrated salt, hydrogen bonding

## Abstract

In the structure of the title hydrated salt, NH_4_^+^·C_8_H_5_Cl_2_O_3_^−^·0.5H_2_O, where the anion derives from (3,5-di­chloro­phen­oxy)acetic acid, the ammonium cation is involved in extensive N—H⋯O hydrogen bonding with both carboxyl­ate and ether O-atom acceptors giving sheet structures lying parallel to (100). The water mol­ecule of solvation lies on a crystallographic twofold rotation axis and is involved in intra-sheet O—H⋯O_carboxyl­ate_ hydrogen-bonding inter­actions. In the anion, the oxoacetate side chain assumes an *antiperiplanar* conformation with the defining C—O—C—C torsion angle = −171.33 (15)°.

## Related literature   

For background on the phen­oxy­acetic acid herbicides, see: Zumdahl (2010 ▸). For examples of structures of a tryptaminium salt and a co-crystalline adduct with (3,5-di­chloro­phen­oxy)acetic acid, see: Smith & Lynch (2015 ▸); Lynch *et al.* (2003 ▸). For the structures of ammonium salts of other phen­oxy­acetic acids, see: Liu *et al.* (2009 ▸); Smith (2014 ▸).
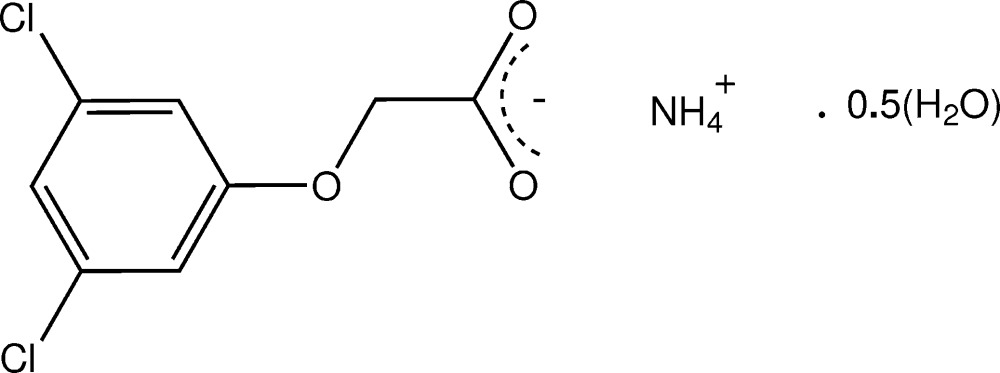


## Experimental   

### Crystal data  

NH_4_^+^·C_8_H_5_Cl_2_O_3_^−^·0.5H_2_O
*M* *_r_* = 247.07
Monoclinic, 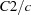

*a* = 39.818 (3) Å
*b* = 4.3440 (4) Å
*c* = 12.7211 (8) Å
β = 98.098 (5)°
*V* = 2178.4 (3) Å^3^
*Z* = 8
Mo *K*α radiation
μ = 0.58 mm^−1^
*T* = 200 K
0.40 × 0.12 × 0.05 mm

### Data collection  

Oxford Diffraction Gemini-S CCD-detector diffractometer
Absorption correction: multi-scan (*CrysAlis PRO*; Agilent, 2013 ▸) *T*_min_ = 0.948, *T*_max_ = 0.980
6680 measured reflections
2146 independent reflections
1832 reflections with *I* > 2σ(*I*)
*R*_int_ = 0.026

### Refinement  

*R*[*F* ^2^ > 2σ(*F* ^2^)] = 0.034
*wR*(*F* ^2^) = 0.084
*S* = 1.08
2146 reflections
147 parameters
5 restraints
H atoms treated by a mixture of independent and constrained refinement
Δρ_max_ = 0.21 e Å^−3^
Δρ_min_ = −0.28 e Å^−3^

### 

Data collection: *CrysAlis PRO* (Agilent, 2013 ▸); cell refinement: *CrysAlis PRO*; data reduction: *CrysAlis PRO*; program(s) used to solve structure: *SIR92* (Altomare *et al.*, 1993 ▸); program(s) used to refine structure: *SHELXL97* (Sheldrick, 2008 ▸) within *WinGX* (Farrugia, 2012 ▸); molecular graphics: *PLATON* (Spek, 2009 ▸); software used to prepare material for publication: *PLATON*.

## Supplementary Material

Crystal structure: contains datablock(s) global, I. DOI: 10.1107/S2056989015016345/nk2232sup1.cif

Structure factors: contains datablock(s) I. DOI: 10.1107/S2056989015016345/nk2232Isup2.hkl

Supporting information file. DOI: 10.1107/S2056989015016345/nk2232Isup3.cml

**Fig. 1.**
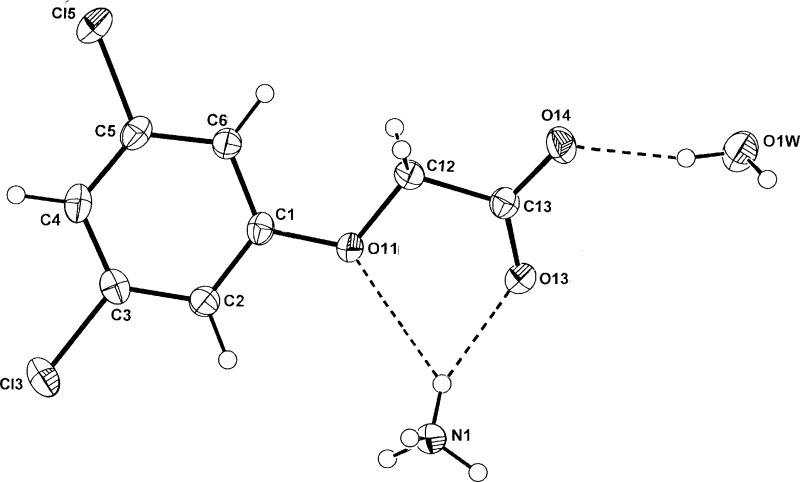
The molecular configuration and atom-numbering scheme for the title hemi-hydrate salt, with non-H atoms shown as 40% probability ellipsoids. The water molecule lies on a twofold rotation axis and inter-species hydrogen bonds are shown as dashed lines.

**Fig. 2.**
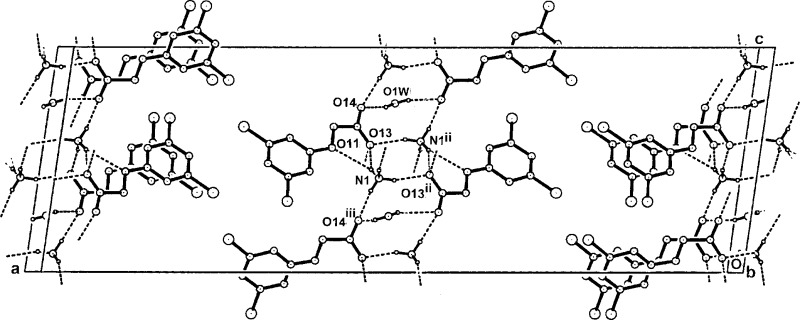
The two-dimensional sheet structure viewed along the b axis, with intramolecular hydrogen bonds shown as dashed lines. For symmetry codes, see Table 1.

. DOI: 10.1107/S2056989015016345/nk2232fig1.tif
The mol­ecular configuration and atom-numbering scheme for the title hemi-hydrate salt, with non-H atoms shown as 40% probability ellipsoids. The water mol­ecule lies on a twofold rotation axis and inter-species hydrogen bonds are shown as dashed lines.

b . DOI: 10.1107/S2056989015016345/nk2232fig2.tif
The two-dimensional sheet structure viewed along the *b* axis, with intra­molecular hydrogen bonds shown as dashed lines. For symmetry codes, see Table 1.

CCDC reference: 1421868

Additional supporting information:  crystallographic information; 3D view; checkCIF report

## Figures and Tables

**Table 1:** Hydrogen-bond geometry (, )

*D*H*A*	*D*H	H*A*	*D* *A*	*D*H*A*
O1*W*H1*W*O14	0.85(2)	1.99(2)	2.822(2)	166(2)
N1H11O11	0.89(2)	2.50(2)	3.137(2)	129(2)
N1H11O13	0.89(2)	1.99(2)	2.811(2)	153(2)
N1H12O13^i^	0.88(2)	2.00(2)	2.862(2)	164(2)
N1H13O14^ii^	0.85(2)	2.03(2)	2.840(2)	161(2)
N1H14O13^iii^	0.90(2)	2.03(2)	2.894(2)	161(2)

Symmetry codes: (i) 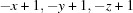; (ii) 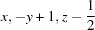; (iii) 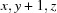.

## supplementary crystallographic information

### S1. Comment

The phenoxy acid (3,5-dichlorophenoxy)acetic acid (3,5-D) is the isomer of the herbicidally active (2,4-dichlorophenoxy)acetic acid (2,4-D) (Zumdahl, 2010). However, unlike 2,4-D the crystallographic literature for 3,5-D is very sparse, comprising only two entries in the Cambridge Structural Database, a 2:1 cocrystal adduct with 4,4'-bipyridine (Lynch *et al.*, 2003) and a tryptaminium salt (Smith & Lynch, 2015). The ammonium salt of 3,5-D, NH_4_^+^ C_8_H_5_Cl_2_O_3_·0.5(H_2_O), was prepared and the structure is reported herein.

In the title salt (Fig. 1), the ammonium cation is involved in extensive N—H···O hydrogen bonding with both carboxyl and ether O-atom acceptors (Table 1), giving two-dimensional sheet structures lying parallel to (100). (Fig. 2). Among these interactions is a centrosymmetric *R*^4^_4_(8) motif conjoined with *R*^4^_4_(12) and *R*^2^_1_(5) motifs, the last one three-centre asymmetric, involving carboxyl and ether O-atom acceptors. The water molecule of solvation (O1*W*) lies on a crystallographic twofold rotation axis and is involved in intra-sheet *O*—*H*···O_carboxyl_ hydrogen-bonding interactions. In this respect, the structure is similar to that of the two-dimensional ammonium salts of the isomeric 2,4-D (Liu *et al.*, 2009) and (4-chloro-2-methylphenoxy)acetic acid (the herbicide MCPA) (Smith, 2014) (both hemihydrates).

The 3,5-DCPA anion is esentially planar with torsion angles C2—C1—O11—C12, C1—O11—C12—C13 and O11—C12—C13—O14 of -175.05 (16), -171.33 (15) and -172.65 (15)° (*antiperiplanar*), of which the defining angle is the second value (about O11—C12). Although the structure of the parent acid (3,5-D) is not known, the value for the title salt is similar to the one found in the tryptaminium salt [-165.5 (3)°] (Smith & Lynch, 2015) and in the ammonium salts of 2,4-D [171.61 (8)°] (Liu *et al.*, 2009) and MCPA [-173.34 (14)°] (Smith, 2014). However, it contrasts with that of the 2:1 adduct of 3,5-D with 4,4'-bipyridine (Lynch *et al.*, 2003) [71.6 (3)°] (*synclinal*).

### S2. Experimental

The title compound was synthesized by adding 1 *M* aqueous ammonia solution dropwise to 10 ml of a solution containing 100 mg of (3,5-dichlorophenoxy)acetic acid in 50% ethanol/water. Room temperature evaporation of the solution gave colourless crystal plates of the title salt from which a specimen was cleaved for the X-ray analysis.

### S3. Refinement

Hydrogen atoms of the hemi-water molecule and the ammonium group were located in a difference-Fourier synthesis and were allowed to ride in the refinement with bond distance restraints O—H = 0.90±0.02 Å and N—H = 0.88±0.02 Å and with *U*_iso_(H) = 1.5*U*_eq_(O) or 1.2*U*_eq_(N). All other H atoms were included at calculated sites and allowed to ride with *U*_iso_(H) = 1.2*U*_eq_(C).

### Crystal data

**Table:**

NH_4_^+^·C_8_H_5_Cl_2_O_3_^−^·0.5H_2_O	*F*(000) = 1016
*M**_r_* = 247.07	*D*_x_ = 1.507 Mg m^−^^3^
Monoclinic, *C*2/*c*	Mo *K*α radiation, λ = 0.71073 Å
Hall symbol: -C 2yc	Cell parameters from 2066 reflections
*a* = 39.818 (3) Å	θ = 4.1–28.7°
*b* = 4.3440 (4) Å	µ = 0.58 mm^−^^1^
*c* = 12.7211 (8) Å	*T* = 200 K
β = 98.098 (5)°	Prism, colourless
*V* = 2178.4 (3) Å^3^	0.40 × 0.12 × 0.05 mm
*Z* = 8	

### Data collection

**Table:**

Oxford Diffraction Gemini-S CCD-detector diffractometer	2146 independent reflections
Radiation source: Enhance (Mo) X-ray source	1832 reflections with *I* > 2σ(*I*)
Graphite monochromator	*R*_int_ = 0.026
Detector resolution: 16.077 pixels mm^-1^	θ_max_ = 26.0°, θ_min_ = 3.1°
ω scans	*h* = −48→39
Absorption correction: multi-scan (*CrysAlis PRO*; Agilent, 2013)	*k* = −3→5
*T*_min_ = 0.948, *T*_max_ = 0.980	*l* = −15→15
6680 measured reflections	

### Refinement

**Table:**

Refinement on *F*^2^	Primary atom site location: structure-invariant direct methods
Least-squares matrix: full	Secondary atom site location: difference Fourier map
*R*[*F*^2^ > 2σ(*F*^2^)] = 0.034	Hydrogen site location: inferred from neighbouring sites
*wR*(*F*^2^) = 0.084	H atoms treated by a mixture of independent and constrained refinement
*S* = 1.08	*w* = 1/[σ^2^(*F*_o_^2^) + (0.034*P*)^2^ + 1.220*P*] where *P* = (*F*_o_^2^ + 2*F*_c_^2^)/3
2146 reflections	(Δ/σ)_max_ = 0.002
147 parameters	Δρ_max_ = 0.21 e Å^−^^3^
5 restraints	Δρ_min_ = −0.28 e Å^−^^3^

### Special details

**Table:**

Geometry. Bond distances, angles *etc*. have been calculated using the rounded fractional coordinates. All su's are estimated from the variances of the (full) variance-covariance matrix. The cell e.s.d.'s are taken into account in the estimation of distances, angles and torsion angles
Refinement. Refinement of *F*^2^ against ALL reflections. The weighted *R*-factor *wR* and goodness of fit *S* are based on *F*^2^, conventional *R*-factors *R* are based on *F*, with *F* set to zero for negative *F*^2^. The threshold expression of *F*^2^ > σ(*F*^2^) is used only for calculating *R*-factors(gt) *etc*. and is not relevant to the choice of reflections for refinement. *R*-factors based on *F*^2^ are statistically about twice as large as those based on *F*, and *R*- factors based on ALL data will be even larger.

### Fractional atomic coordinates and isotropic or equivalent isotropic displacement parameters (Å^2^)

**Table:**

	*x*	*y*	*z*	*U*_iso_*/*U*_eq_	
Cl3	0.66552 (1)	1.11332 (13)	0.31644 (4)	0.0401 (2)	
Cl5	0.72836 (1)	0.45912 (16)	0.64617 (5)	0.0517 (2)	
O11	0.59904 (3)	0.5049 (3)	0.55326 (10)	0.0340 (4)	
O13	0.53963 (3)	0.2408 (3)	0.56560 (10)	0.0325 (4)	
O14	0.55624 (4)	0.0775 (3)	0.73120 (11)	0.0384 (5)	
C1	0.63101 (4)	0.5896 (4)	0.53513 (14)	0.0268 (5)	
C2	0.63218 (5)	0.7850 (4)	0.44879 (14)	0.0282 (6)	
C3	0.66354 (5)	0.8742 (4)	0.42535 (14)	0.0288 (6)	
C4	0.69369 (5)	0.7798 (5)	0.48405 (15)	0.0338 (6)	
C5	0.69140 (5)	0.5888 (5)	0.56904 (15)	0.0322 (6)	
C6	0.66063 (4)	0.4896 (5)	0.59619 (14)	0.0280 (6)	
C12	0.59684 (5)	0.3252 (5)	0.64620 (14)	0.0314 (6)	
C13	0.56109 (4)	0.2087 (4)	0.64708 (14)	0.0274 (6)	
O1W	0.50000	−0.3034 (5)	0.75000	0.0456 (8)	
N1	0.53131 (4)	0.7283 (5)	0.41888 (14)	0.0332 (6)	
H2	0.61190	0.85480	0.40720	0.0340*	
H4	0.71500	0.84390	0.46650	0.0410*	
H6	0.65990	0.35670	0.65510	0.0340*	
H121	0.60370	0.45230	0.71020	0.0380*	
H122	0.61260	0.14830	0.64820	0.0380*	
H1W	0.5153 (5)	−0.185 (5)	0.7335 (17)	0.0400*	
H11	0.5403 (5)	0.568 (4)	0.4560 (15)	0.0320*	
H12	0.5089 (4)	0.732 (5)	0.4100 (15)	0.0320*	
H13	0.5406 (5)	0.744 (5)	0.3632 (13)	0.0320*	
H14	0.5370 (5)	0.904 (4)	0.4539 (15)	0.0320*	

### Atomic displacement parameters (Å^2^)

**Table:**

	*U*^11^	*U*^22^	*U*^33^	*U*^12^	*U*^13^	*U*^23^
Cl3	0.0507 (3)	0.0386 (3)	0.0329 (3)	−0.0105 (2)	0.0122 (2)	0.0034 (2)
Cl5	0.0240 (3)	0.0735 (5)	0.0549 (4)	−0.0024 (3)	−0.0035 (2)	0.0113 (3)
O11	0.0217 (7)	0.0486 (9)	0.0323 (7)	−0.0008 (6)	0.0055 (5)	0.0128 (6)
O13	0.0252 (7)	0.0387 (8)	0.0330 (7)	−0.0004 (6)	0.0020 (6)	0.0037 (6)
O14	0.0370 (8)	0.0504 (9)	0.0298 (7)	−0.0067 (7)	0.0112 (6)	0.0059 (7)
C1	0.0240 (9)	0.0309 (10)	0.0264 (9)	−0.0031 (8)	0.0066 (7)	−0.0046 (8)
C2	0.0278 (10)	0.0319 (11)	0.0250 (9)	0.0002 (8)	0.0040 (7)	−0.0012 (8)
C3	0.0361 (11)	0.0264 (10)	0.0253 (9)	−0.0054 (8)	0.0096 (8)	−0.0051 (8)
C4	0.0283 (10)	0.0386 (12)	0.0361 (11)	−0.0098 (9)	0.0098 (8)	−0.0058 (9)
C5	0.0239 (10)	0.0391 (12)	0.0326 (11)	−0.0020 (9)	0.0002 (8)	−0.0054 (9)
C6	0.0257 (10)	0.0334 (11)	0.0250 (9)	−0.0020 (8)	0.0035 (7)	−0.0001 (8)
C12	0.0280 (10)	0.0420 (12)	0.0239 (9)	−0.0028 (9)	0.0024 (7)	0.0062 (8)
C13	0.0265 (10)	0.0294 (10)	0.0277 (10)	0.0032 (8)	0.0088 (8)	−0.0016 (8)
O1W	0.0348 (12)	0.0361 (13)	0.0660 (15)	0.0000	0.0073 (11)	0.0000
N1	0.0282 (9)	0.0392 (11)	0.0325 (10)	0.0027 (8)	0.0050 (7)	0.0076 (8)

### Geometric parameters (Å, º)

**Table:**

Cl3—C3	1.7423 (18)	C1—C6	1.387 (2)
Cl5—C5	1.743 (2)	C1—C2	1.394 (2)
O11—C1	1.375 (2)	C2—C3	1.380 (3)
O11—C12	1.430 (2)	C3—C4	1.384 (3)
O13—C13	1.255 (2)	C4—C5	1.376 (3)
O14—C13	1.251 (2)	C5—C6	1.388 (3)
O1W—H1W^i^	0.85 (2)	C12—C13	1.512 (3)
O1W—H1W	0.85 (2)	C2—H2	0.9500
N1—H13	0.847 (18)	C4—H4	0.9500
N1—H12	0.884 (16)	C6—H6	0.9500
N1—H11	0.888 (18)	C12—H122	0.9900
N1—H14	0.897 (18)	C12—H121	0.9900
			
C1—O11—C12	116.79 (14)	C4—C5—C6	122.78 (18)
H1W—O1W—H1W^i^	105 (2)	C1—C6—C5	118.31 (17)
H12—N1—H13	116.4 (18)	O11—C12—C13	110.87 (15)
H12—N1—H14	103.2 (19)	O13—C13—C12	119.27 (15)
H11—N1—H12	114.0 (19)	O13—C13—O14	126.01 (16)
H11—N1—H13	108.5 (19)	O14—C13—C12	114.68 (16)
H13—N1—H14	103.7 (19)	C3—C2—H2	121.00
H11—N1—H14	110.4 (17)	C1—C2—H2	121.00
C2—C1—C6	120.77 (16)	C5—C4—H4	121.00
O11—C1—C6	123.80 (16)	C3—C4—H4	122.00
O11—C1—C2	115.43 (15)	C1—C6—H6	121.00
C1—C2—C3	118.23 (17)	C5—C6—H6	121.00
Cl3—C3—C2	118.89 (14)	O11—C12—H121	109.00
Cl3—C3—C4	118.22 (15)	O11—C12—H122	109.00
C2—C3—C4	122.89 (17)	C13—C12—H121	109.00
C3—C4—C5	117.02 (18)	C13—C12—H122	110.00
Cl5—C5—C4	119.54 (16)	H121—C12—H122	108.00
Cl5—C5—C6	117.68 (15)		
			
C12—O11—C1—C2	−175.05 (16)	Cl3—C3—C4—C5	−179.42 (15)
C12—O11—C1—C6	5.6 (3)	C2—C3—C4—C5	0.0 (3)
C1—O11—C12—C13	−171.33 (15)	C3—C4—C5—Cl5	179.67 (15)
O11—C1—C2—C3	−178.93 (15)	C3—C4—C5—C6	0.5 (3)
C6—C1—C2—C3	0.5 (3)	Cl5—C5—C6—C1	−179.64 (15)
O11—C1—C6—C5	179.27 (17)	C4—C5—C6—C1	−0.4 (3)
C2—C1—C6—C5	−0.1 (3)	O11—C12—C13—O13	9.5 (2)
C1—C2—C3—Cl3	178.97 (13)	O11—C12—C13—O14	−172.65 (15)
C1—C2—C3—C4	−0.4 (3)		

Symmetry code: (i) −*x*+1, *y*, −*z*+3/2.

### Hydrogen-bond geometry (Å, º)

**Table:**

*D*—H···*A*	*D*—H	H···*A*	*D*···*A*	*D*—H···*A*
O1*W*—H1*W*···O14	0.85 (2)	1.99 (2)	2.822 (2)	166 (2)
N1—H11···O11	0.89 (2)	2.50 (2)	3.137 (2)	129 (2)
N1—H11···O13	0.89 (2)	1.99 (2)	2.811 (2)	153 (2)
N1—H12···O13^ii^	0.88 (2)	2.00 (2)	2.862 (2)	164 (2)
N1—H13···O14^iii^	0.85 (2)	2.03 (2)	2.840 (2)	161 (2)
N1—H14···O13^iv^	0.90 (2)	2.03 (2)	2.894 (2)	161 (2)

Symmetry codes: (ii) −*x*+1, −*y*+1, −*z*+1; (iii) *x*, −*y*+1, *z*−1/2; (iv) *x*, *y*+1, *z*.
